# Trophic discrimination factor of nitrogen isotopes within amino acids in the dobsonfly *Protohermes grandis* (Megaloptera: Corydalidae) larvae in a controlled feeding experiment

**DOI:** 10.1002/ece3.2728

**Published:** 2017-03-12

**Authors:** Naoto F. Ishikawa, Fumio Hayashi, Yoko Sasaki, Yoshito Chikaraishi, Naohiko Ohkouchi

**Affiliations:** ^1^ Japan Agency for Marine‐Earth Science and TechnologyYokosukaKanagawaJapan; ^2^ Department of BiologyTokyo Metropolitan UniversityHachiojiTokyoJapan; ^3^ Present address: ETH ZürichSonneggstrasse 58092ZürichSwitzerland; ^4^ Present address: Institute of Low Temperature ScienceHokkaido UniversityKita 19, Nishi 8, Kita‐kuSapporo060‐0819Japan

**Keywords:** compound‐specific isotope analysis of amino acids, deamination, starvation, top predator, trophic position

## Abstract

The trophic discrimination factor (TDF) of nitrogen isotopes (^15^N/^14^N) within amino acids, between a stream‐dwelling dobsonfly larva (*Protohermes grandis*: Megaloptera; Corydalidae) and its diet (chironomid larvae), was determined in controlled feeding experiments. Last‐instar larvae of *P. grandis* were collected from the Yozawa‐gawa River, central Japan, and reared in the laboratory. After fed to satiation for 1 month, one group of larvae was each fed one living chironomid larva per day for 4 weeks, while a second group was starved for 8 weeks. The larvae were harvested at intervals and the nitrogen isotopic composition of glutamic acid (*δ* ^15^N_Glu_) and phenylalanine (*δ* ^15^N_Phe_) were determined to calculate TDF. The mean TDF of satiated and starved larvae were 7.1‰ ± 0.5‰ (*n *= 3) and 7.3‰ ± 0.5‰ (*n *=* *5), respectively. Thus, the TDF for *P. grandis* larvae in this study was similar to that reported for other arthropods (approximately 7‰) and was independent of satiation or starvation. A previous study of wild *P. grandis* larvae, based on the *δ* ^15^N_Glu_ and *δ* ^15^N_Phe_ values, estimated its trophic position (TP) as approximately 2.0 ± 0.1 (*n *=* *5), a low value close to that of algivores, although they are generally characterized as carnivores (usually accepted as TP ≥ 3). The TDF for *P. grandis* larvae suggests that their low TPs in nature were caused by incorporation of vascular plant‐derived amino acids (with a different *δ* ^15^N profile from that of algae) and not by an unusually low TDF or by the effects of the satiation/starvation on amino acid metabolism.

## 1 Introduction

The trophic position (TP) of an animal is an important dimension of its ecological niche in a given food web (Chase & Leibold, 2003). Knowledge of the TP of top‐predator animals in a food web is useful to assess the food chain length and is a key factor for understanding ecosystem function (e.g., Vander Zanden & Fetzer, 2007). To accurately and precisely estimate TPs of animals in various ecosystems, the compound‐specific isotope analysis of amino acids (CSIA‐AA) has recently been used (e.g., Bowes & Thorp, 2015; Chikaraishi, Kashiyama, Ogawa, Kitazato, & Ohkouchi, 2007; Chikaraishi et al., 2009; McClelland & Montoya, 2002; Steffan et al., 2013). In amino acid metabolism, glutamic acid is deaminated, leading to enrichment of ^15^N at each trophic level (TL) (e.g., Δ*δ* ^15^N_consumer−diet_ = 8.0‰, McClelland & Montoya, 2002; Chikaraishi et al., 2009). In contrast, phenylalanine retains its amino group because of its low deamination flux during the metabolism, resulting in a negligible enrichment in ^15^N per TL (e.g., Δ*δ* ^15^N_consumer−diet_ = 0.4‰, McClelland & Montoya, 2002; Chikaraishi et al., 2009). Based on this principle, the *δ* ^15^N values of glutamic acid and phenylalanine within a single animal can provide information concerning its TP, using the following equation with an analytical precision (1 σ) of 0.12 units (Chikaraishi et al., 2009):

(1) $$\mathrm{TP}=\frac{\delta^{15}N_{\mathrm{Glu}}-\delta^{15}N_{\mathrm{Phe}}+\beta }{\mathrm{TDF}}+1$$

where *δ* ^15^N_Glu_ and *δ* ^15^N_Phe_ are the *δ* ^15^N values of glutamic acid (as a representative “trophic” amino acid) and phenylalanine (as a representative “source” amino acid), respectively. The constant, β, represents the difference between *δ* ^15^N_Phe_ and *δ* ^15^N_Glu_ in a primary producer at the base of the food chain (i.e., −3.4‰ for algae and cyanobacteria; +8.4‰ for vascular plants; Chikaraishi et al., 2009; Chikaraishi, Ogawa, & Ohkouchi, 2010; Chikaraishi, Ogawa, Doi, & Ohkouchi, 2011). TDF is the trophic discrimination factor (i.e., Δ*δ* ^15^N_Glu_ − Δ*δ* ^15^N_Phe_) at each shift of TL, which has been shown to be universal across various taxonomic groups (Steffan et al., 2013, 2015). Compared with traditional approaches (e.g., *δ* ^15^N analysis of bulk animal tissues), the CSIA‐AA method has significant advantages. In particular, the TP can be estimated from the combined *δ* ^15^N_Glu_ and *δ* ^15^N_Phe_ values within a single organism of interest, and the *δ* ^15^N values of primary producers at the base of food webs are approximately indicated by the *δ* ^15^N_Phe_ values of organisms at any TP in the food webs (e.g., Chikaraishi et al. 2014; Choy, Popp, Hannides, & Drazen, 2015; McCarthy, Benner, Lee, & Fogel, 2007; Popp et al., 2007; Steffan et al., 2015).

CSIA‐AA has been applied to the stream food web analysis in a previous study (Ishikawa et al., 2014). In stream ecosystems, dobsonfly larvae (Megaloptera: Corydalidae) are one of the carnivorous insects that are widely distributed throughout the world. Larvae of *Protohermes grandis* (Thunberg, 1781) are considered to be nearly the top predators in stream macroinvertebrate food webs in Japan, based on their large mandibles and foregut contents (Hayashi, 1988a; Takemon, 2005). However, CSIA‐AA estimated the TP of *P. grandis* larvae as 2.0 ± 0.1 (*n *=* *5, based on β = −3.4‰), considerably lower than the value of TP ≥ 3 expected from its biological and functional position as a carnivorous animal (Ishikawa et al., 2014). This could be explained by (1) species‐specific unusually low TDF, (2) reduction in the TDF associated with prolonged starvation inherent in their lifestyle, or (3) increased β value caused by incorporation of vascular plant‐derived amino acids into the basal resources of the stream food web.

With respect to the first hypothesis, if the TDF of *P. grandis* larva is lower than that reported in the literature for other organisms (e.g., 7.6‰, Chikaraishi et al., 2009), the TP of *P. grandis* larvae would be lower than expected. In relation to the second, the resource availability in stream ecosystems is typically low compared with coastal marine and terrestrial ecosystems and the foreguts of wild *P. grandis* larvae are often empty (Hayashi, 1988a). This implies that *P. grandis* larvae may, at times, be at various stages of starvation. Thus, their metabolism might be specifically adapted to such starvation. Third, incorporation of amino acids derived from vascular plants into the diet animals of *P. grandis* could increase the β value in Equation (1) from −3.4‰ (used for algal‐based food webs) toward +8.4‰ (used for vascular plant‐based food webs), resulting in an apparent underestimation of TP for *P. grandis*. In this case, the mixing proportion of amino acids derived from aquatic autotrophs and vascular plants to the basal diet resources of *P. grandis* must be calculated to find the TP accurately. To examine the above hypotheses (1) and (2), in this study we reared wild‐caught *P. grandis* larvae in controlled feeding experiments in the laboratory, under satiated and starved conditions, and determined the *δ* ^15^N_Glu_ and *δ* ^15^N_Phe_ values to calculate TDF.

## 2 Materials and methods

### 2.1 Sample collection and laboratory experiments

**Figure 1:**
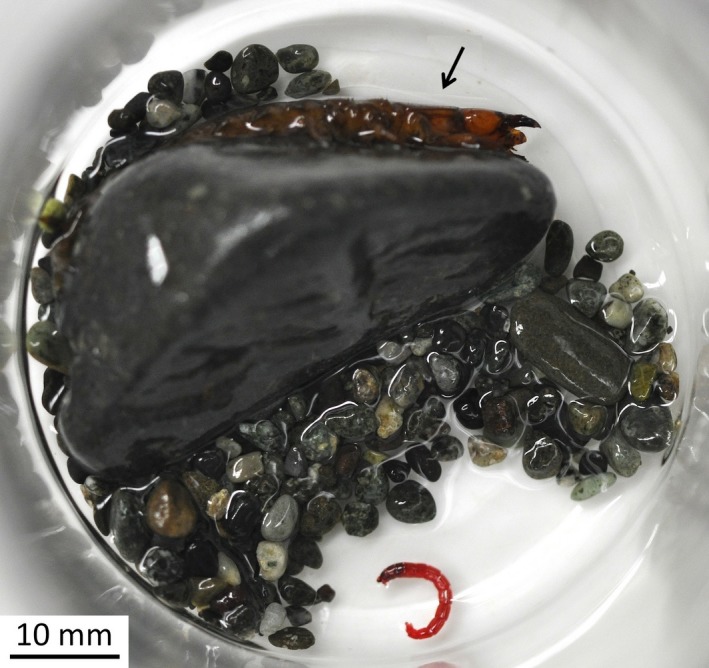
Rearing conditions (dorsal view) of *Protohermes grandis* larvae provided with one live chironomid larva per day. Small stones on the glass vessel bottom (arrow) provide a refuge for the *P. grandis* larva. Water depth was kept quite shallow (<5 mm) to avoid oxygen depletion. At night, the larva moved around and fed on the prey

We collected last‐instar larvae of *P. grandis* from the Yozawa‐gawa River (35°44′N, 139°11′E), a tributary of the Tama‐gawa River, central Japan, on 01 October 2014. We used last‐instar larvae in this study because the larval period is 2–3 years in the field and it is impossible to rear the larva from the first instar to the last instar in the laboratory (Hayashi, 1988b). The last‐instar larvae stay in a stream for about 8 months (from autumn to the following spring) and increase greatly in body mass before pupating. Therefore, we chose this stage of larvae that just molted to the last instar in early autumn. Great care was taken to prevent injury caused by the larvae biting each other. Larvae were placed into individual plastic vessels for transfer to the laboratory. As a wild reference, one individual was immediately frozen until the isotope measurement. Other larvae were placed individually in glass vessels (62 mm diameter × 82 mm) with several stones as a refuge (Figure 1). Well‐aerated tap water was supplied, not exceeding 5 mm in depth, and replaced daily. The rearing vessels were kept in an incubator at constant temperature of 20 ± 1°C, and 12:12‐h L:D light cycle.

Larvae of *P. grandis* were fed a single living chironomid larva, *Propsilocerus akamusi* (Tokunaga, 1938), per day for a month, to establish uniform *δ* ^15^N values among individuals. Chironomid larvae are one of the main prey items for wild *P. grandis* larvae (Hayashi, 1988a). Two clumps of the last‐instar larvae of *P. akamushi* were purchased from a fishing‐tackle shop and were kept alive at a low basal metabolic rate at 4°C in a refrigerator during the experimental period. This chironomid has a univoltine life cycle, and the last‐instar larvae enter a long summer diapause from May to September (Yamagishi & Fukuhara, 1971). Although the *δ* ^15^N value of the chironomid larvae was predicted to change during the experimental period, this variation should not theoretically propagate to their TP estimates as long as all the cohorts had been fed the same diet. This is because the *δ* ^15^N value of trophic amino acid (i.e., glutamic acid) is subtracted by that of source amino acid (i.e., phenylalanine) in Equation (1) (Chikaraishi et al., 2009), with the assumption that isotopic fluctuations occur in parallel among amino acids. To assess the variability of both the *δ* ^15^N values and TP estimates in the diet of *P. grandis* during the experimental period, three chironomid larvae were randomly chosen for the isotope measurement.

After the 1‐month period of satiation, one individual of *P. grandis* larva was chosen as an initial representative and was frozen. The remaining larvae were divided into two groups: “Satiated” larvae were continuously reared with one living chironomid larva per day (*n *=* *3 including the initial individual), and “starved” larvae were left unfed (*n *=* *5). One larva was harvested and frozen from the satiated group at 2 and 4 weeks after the experiment started and from the starved group after 1, 2, 4, 6, and 8 weeks. One pupa of *P. grandis* that had been reared with one living chironomid larva per day for 14 weeks of its larval period was also used for the isotope measurement. Isotopic variability among *P. grandis* individuals should reflect that among the chironomid larvae, and the isotopic composition of amino acids in the three representative chironomid larvae was determined. Therefore, we analyzed one individual of the *P. grandis* larva at each time point.

### 2.2 Isotope measurement

Single forelegs of *P. grandis* larvae and pupa were dissected to obtain muscle samples for the isotope measurement, while whole bodies of the chironomid larvae were used for the measurement. For CSIA‐AA, amino acids in all samples were purified by HCl hydrolysis followed by *N*‐pivaloyl/isopropyl addition, according to the improved procedures of Chikaraishi et al. (2007). The *δ* ^15^N values of amino acids were determined by the modified method of Chikaraishi, Takano, Ogawa, and Ohkouchi (2010). Isotopic reference mixtures of 10 amino acids (*δ* ^15^N ranging from −26.6‰ to +45.7‰; Indiana University, Bloomington, USA, and SI Science Co., Sugito‐machi, Japan) were analyzed every five or six samples to confirm the reproducibility of the isotope measurements. Analytical errors (1 σ) of the standards were better than 0.5‰, with a minimum sample quantity of 0.5 nmol N.

TPs of the *P. grandis* and chironomid larvae were estimated using Equation (1). β value was set at −3.4‰ (used for algal‐based food webs). The TDF was calculated using the following equation:

(2) $$\mathrm{TDF}=\left(\delta^{15}N_{\mathrm{GluPro}}\;-\;\delta^{15}N_{\mathrm{GluChi}}\right)-\left(\delta^{15}N_{\mathrm{PhePro}}-\delta^{15}N_{\mathrm{PheChi}}\right)$$

where *δ* ^15^N_GluPro_ and *δ* ^15^N_PhePro_ are *δ* ^15^N_Glu_ and *δ* ^15^N_Phe_ values, respectively, of *P. grandis* larvae, and *δ* ^15^N_GluChi_ and *δ* ^15^N_PheChi_ are mean *δ* ^15^N_Glu_ and *δ* ^15^N_Phe_ values, respectively, of chironomid larvae (*n *=* *3). Two‐way ANOVA was used to test the effects of treatment (i.e., satiated vs. starved groups) and time (i.e., experimental period) on the TP of *P. grandis* larva.

## 3 Results

The estimated TP of the wild *P. grandis* larva (the reference individual) from the Yozawa‐gawa River, based on the *δ* ^15^N values of amino acids, was 2.2 (Table 1), although the natural variability in this site was unknown. The estimate was consistent with the TP observed in wild *P. grandis* larvae from different locations, reported in a previous study (2.0 ± 0.1, *n *=* *5, Ishikawa et al., 2014). However, after 1‐month satiation (pre‐experiment), the estimated TP of *P. grandis* larvae was elevated to 2.7, a value approximately one‐unit higher than that of chironomid larvae (TP = 1.8 ± 0.1, *n *=* *3), the only diet available for *P. grandis* in the experiment (Table 1). Although isotope variability among individuals was not available due to the lack of replications, the TP of the *P. grandis* larvae satiated for 1 month was quite consistent with that of the pupa (TP = 2.8, satiated for 14 weeks) (Table 1).

**Table 1:** Values of *δ* ^15^N (‰) of amino acids for the larvae and pupa of *Protohermes grandis* and their experimental diet (living chironomid larvae)

Specimen	Group	Period (weeks)	Ala	Gly	Val	Leu	Ile	Glu	Phe	TP
*P. grandis* (larva)	Wild	–	9.1	2.0	16.8	ND	ND	10.5	−2.2	2.2
	Satiated	0	6.9	−2.5	6.8	3.5	8.5	15.0	−1.6	2.7
	Satiated	2	7.9	2.5	15.6	4.7	15.0	13.2	−2.6	2.6
	Satiated	4	11.1	2.2	15.8	7.2	15.1	14.3	−2.3	2.7
	Starved	1	11.6	−0.7	13.0	ND	16.6	14.7	−1.7	2.7
	Starved	2	ND	−1.4	15.9	7.0	17.9	13.3	−2.4	2.6
	Starved	4	9.3	0.0	13.0	6.6	15.9	14.7	−2.4	2.8
	Starved	6	9.9	1.2	15.9	10.2	15.4	14.7	−1.9	2.7
	Starved	8	10.4	0.9	13.5	6.1	19.4	14.8	−2.0	2.8
*P. grandis* (pupa)	Satiated	14	11.4	−11.8	13.2	7.1	8.0	12.9	−4.0	2.8
Chironomid (larva)	Diet	–	8.3	−1.7	12.1	4.1	9.3	9.0	0.4	1.7
	Diet	–	2.1	−14.3	2.4	−3.3	3.0	3.6	−6.5	1.9
	Diet	–	8.9	−8.5	10.7	4.3	7.8	8.4	−0.6	1.7

The wild group was larva collected from the field. Satiated and starved groups refer to larvae that were fed chironomid larvae (1 day^−1^) and unfed, respectively. After the 1‐month preconditioning (one chironomid per day), the values of *δ* ^15^N (‰) were analyzed for the larvae at 0, 2, and 4 weeks in the satiated group and at 1, 2, 4, 6, and 8 weeks in the starved group. Ala, alanine; Gly, glycine; Val, valine; Leu, leucine; Ile, isoleucine; Glu, glutamic acid; Phe, phenylalanine; TP, trophic position (see Equation (1)). ND, not determined.

There was a large variation in the *δ* ^15^N value within the same amino acids among individual chironomid larvae (e.g., 7.0‰ ± 3.0‰ in *δ* ^15^N_Glu_, −2.2‰ ± 3.7‰ in *δ* ^15^N_Phe_, Table 1). This may reflect heterogeneity of *δ* ^15^N values among individuals and/or their state of arrested metabolism during storage at 4°C. Although these factors could have changed the amino acid metabolism during the feeding experiments, the *δ* ^15^N variation was generally parallel between glutamic acid and phenylalanine, resulting in little variation in the TP for the chironomid larva (1 σ of 0.1, *n *=* *3, Table 1). The *δ* ^15^N_Phe_ values of satiated *P. grandis* larvae and pupa ranged from −4.0‰ to −1.6‰, which were within the range of *δ* ^15^N_Phe_ values of the chironomid larva. These values are consistent with little trophic enrichment of ^15^N for phenylalanine between consumer (i.e., *P. grandis* larvae and pupa) and its diet (i.e., chironomid larvae).

The TP of *P. grandis* larvae did not significantly differ among treatments and time (range: 2.6–2.8; satiated group: 2.7 ± 0.1, *n *=* *3; starved group: 2.7 ± 0.1, *n *=* *5, two‐way ANOVA, treatment: *F *=* *0.006, *p *=* *.94; time: *F *=* *1.013, *p *=* *.37; treatment × time: *F *=* *0.277, *p *=* *.63, Table 1), although there was also a large variation in both the *δ* ^15^N_Glu_ and *δ* ^15^N_Phe_ values for *P. grandis*. These results confirmed that isotopic fluctuations occur almost in parallel among amino acids and that a month of satiation prior to the main experiment established the uniform TP of *P. grandis* larvae.

The mean TDF values of satiated and starved *P. grandis* larvae were 7.1‰ ± 0.5‰ (*n *=* *3) and 7.3‰ ± 0.5‰ (*n *=* *5), respectively (Table 2). These values are similar to those reported for other insects in feeding experiments on terrestrial food chains (7.6‰ ± 0.1‰, Steffan et al., 2013), for white shrimps fed mixed pellets of microalgae and squid (6.5‰, Downs, Popp, & Holl, 2014 and references therein), and for an aquatic vertebrate (tadpoles) fed dried (dead) bloodworms (7.5‰ ± 0.4‰, Chikaraishi, Steffan, Takano, & Ohkouchi, 2015) (Table 2). The TDF of starved *P. grandis* larvae was surprisingly similar to that of satiated larvae. This clearly indicates that *P. grandis* did not exhibit an unusually low TDF during the feeding experiments and that the magnitude of the TDF was independent of the state of satiation or starvation for at least 8 weeks.

**Table 2:** Trophic discrimination factor (TDF) between satiated (*n *=* *3) and starved (*n *=* *5) *Protohermes grandis* larvae and their chironomid diet, calculated using Equation (2) (see text), and comparison with three other systems observed in feeding experiments: a terrestrial insect food chain (apple leaves, apple aphids, hover flies, parasitoids, and hyperparasitoids); white shrimps (*Litopenaeus vannamei*) fed a mixture of microalgae and squid; and tadpoles of *Bufo japonica* fed dried (dead) bloodworms

System	TDF	*SD*	Reference
Satiated *P. grandis* larva (fed with chironomid larvae)	7.1	0.5	This study
Starved *P. grandis* larva (unfed following 1‐month satiation with chironomid larvae)	7.3	0.5	This study
Terrestrial insect food chain	7.6	0.1	Steffan et al. (2013)
Shrimps fed with microalgae and squids	6.5	–	Downs et al. (2014) and references therein
Tadpoles fed with dried bloodworms	7.5	0.4	Chikaraishi et al. (2015)

## 4 Discussion

Ecologically unrealistically low TP values estimated for wild *P. grandis* larvae in a previous study (Ishikawa et al., 2014) could be explained by one of the following three hypotheses: (1) species‐specific unusually low TDF; (2) an effect of starvation on TDF; or (3) high β value caused by incorporation of vascular plant‐derived amino acids into the stream food web. However, the hypotheses (1) and (2) are rejected by the feeding experiments in this study, although the quality and quantity of the diet differed from that in the natural settings. In addition to the chironimid larvae, various kinds of macroinvertebrates have been found in the foreguts of the wild *P. grandis* larvae, but in many cases, their foreguts are empty (Hayashi, 1988a). The third hypothesis is a possible explanation, supported by both the observed TDF in this study and biological observations in the literature. Larvae of *P. grandis* are generalist predators that feed on a range of aquatic animals, including algivorous and detritivorous insects, and other predators (Hayashi, 1988a). Detritivores may feed on the remains of vascular plants (e.g., leaf litter) in stream environments, transferring amino acids from vascular plants to *P. grandis* larva. If a β value of +8.4‰ (used for vascular plant‐based food webs) was applied in Equation (1), the TP of the wild *P. grandis* larva would be estimated as 3.8. Therefore, in case that the “real” TP of the wild *P. grandis* larva is ≥3, which is between 2.2 (i.e., β = −3.4‰) and 3.8 (i.e., β = +8.4‰), a hypothetical mixing model would estimate that ≥49% of amino acids in *P. grandis* larva are derived from vascular plants.

The trophic discrimination of nitrogen isotopes is attributable to the isotopic fractionation associated with deamination of amino acids in animals (Chikaraishi et al., 2007; Ohkouchi, Ogawa, Chikaraishi, Tanaka, & Wada, 2015). Deamination is a major initial step in the breakdown of trophic amino acids to produce metabolic energy for growth (Bender, 2012), and the balance of input (assimilation) and output (deamination) is theoretically an essential factor to determine the magnitude of the TDF (Chikaraishi et al., 2015; McMahon, Thorrold, Elsdon, & McCarthy, 2015). This isotopic discrimination was empirically supported by an enzymatic experiment that showed that the *δ* ^15^N value of the remaining amino acids was gradually elevated as the deamination flux increased (Macko, Estep, Engel, & Hare, 1986; Miura & Goto, 2012). The fairly constant TDF values observed in previous feeding experiments (Downs et al., 2014; Steffan et al., 2013) suggest that the proportions of deaminated amino acids are almost identical within arthropods that are fully satiated. In other words, an unusual isotopic discrimination is expected if an animal is forced into a state of severe famine, in which the budget of the available amino acids is considerably reduced (Chikaraishi et al., 2015; McMahon et al., 2015). This is because consumers may have to continuously facilitate deamination to obtain metabolic energy even without additional input of amino acids derived from their diets.

However, in this study, we observed no substantial difference in the TDF between satiated and starved *P. grandis* larvae. Furthermore, we found that *P. grandis* larvae tolerate starvation for at least 8 weeks. These observations suggest that the animal overcomes starvation without a substantial increase in output (i.e., metabolic breakdown) of amino acids, although they would need some metabolic energy for homeostasis. This phenomenon leads to several critical questions as follows: Is it possible that, in the wild, animals do not produce metabolic energy from amino acids? If yes, then from where is the metabolic energy derived? With respect to the *P. grandis* larvae, the respiratory quotient (i.e., carbon dioxide emission divided by oxygen consumption in a given time) is typically approximately 0.7, suggesting that they obtain energy in part by combusting storage lipids (Hayashi & Yoshida, 1987). Therefore, we suggest that starved dobsonfly larvae produce metabolic energy from storage lipids or carbohydrates rather than from proteins. These alternative sources serve as an “energetic buffer” against a starvation, resulting in unchanging *δ* ^15^N and TDF values for amino acids because of disuse of amino acids as metabolic fuel. This adaptation to starvation means that ecologists might observe relatively unvarying (or almost constant) TP estimated by using CSIA‐AA in wild animals, even when exposed to changing conditions of satiation and starvation. Thus, starvation‐independent TDF will be key to evaluating the broad applicability of CSIA‐AA methodology to a number of heterogenetic ecosystems.

## Conflict of interest

None declared.
